# Impfquotenmonitoring in Deutschland – gegenwärtiger Stand und Perspektive

**DOI:** 10.1007/s00103-025-04020-w

**Published:** 2025-02-19

**Authors:** Thorsten Rieck, Cornelius Rau, Elisa Wulkotte, Marcel Feig, Constantin Fischer, Ole Wichmann, Annika Steffen

**Affiliations:** 1 Fachgebiet Impfprävention/STIKO, Abteilung für Infektionsepidemiologie, Robert Koch-Institut, Berlin, Deutschland Seestr. 10, 13353; 2 Fachgebiet Fachdaten-Kompetenzzentrum, Abteilung für Methodenentwicklung, Forschungsinfrastruktur und Informationstechnologie, Robert Koch-Institut, Berlin, Deutschland Seestr. 10, 13353

**Keywords:** Impfung, Impfquoten, Monitoring, Datenerhebung, Gesundheitsdaten, Vaccination, Vaccination coverage, Monitoring, Data collection, Health data

## Abstract

Aktuelle und belastbare Daten zum Impfstatus der Bevölkerung sind für die Evaluation von Impfprogrammen unerlässlich. Die systematische Auswertung von Daten der Schuleingangsuntersuchungen (SEU) und Abrechnungsdaten der Kassenärztlichen Vereinigungen (KVen) ist Grundlage des Impfquotenmonitorings in Deutschland. Diese beiden etablierten, effektiven Systeme werden durch Surveys ergänzt, die für besondere Zielgruppen neben Daten zur Impfinanspruchnahme auch Informationen zu Impfakzeptanz und -intention liefern können.

Anhand der SEU- und Abrechnungsdaten können Impfquoten nur mit einem Zeitverzug von 1–2 Jahren berichtet werden. Außerdem sind die Abrechnungsdaten aufgrund zunehmender Diversifizierung von Impfleistungserbringern unvollständig. Die Notwendigkeit eines vollständigen und zeitnahen Impfquotenmonitorings während der Covid-19-Pandemie zeigte die Grenzen dieser Systeme in einer Akutsituation auf. Daher wurde mit dem Digitalen Impfquotenmonitoring (DIM) ein temporäres System zur Erhebung tagesaktueller Covid-19-Impfdaten etabliert, das allen Impfleistungserbringern die Datenübermittlung an das Robert Koch-Institut (RKI) ermöglichte. Mit der Vision eines zeitnahen und vollständigen Impfquotenmonitorings wird die Integration der Impfdatenerhebung in das Deutsche Elektronische Melde- und Informationssystem für den Infektionsschutz (DEMIS) als einheitliche Meldeinfrastruktur vorangetrieben.

Über DEMIS sollen zukünftig pseudonymisierte, einzelfallbasierte Impfdaten zeitnah von allen Impfleistungserbringern an das RKI übermittelt werden. Damit wird es möglich sein, die Umsetzung neuer Impfempfehlungen anhand vervollständigter und hoch aufgelöster Daten mit geringem Zeitverzug zu bewerten und Impfempfehlungen sowie Kommunikationsstrategien im Bedarfsfall schnell anzupassen.

## Abstract (en)

Timely and reliable data on the vaccination status of the population are essential for the evaluation of vaccination programs. The analysis of school entry examination data and health claims data from the Associations of Statutory Health Insurance Physicians is the basis for monitoring the vaccination coverage in Germany. These two established and effective systems are supplemented by surveys among special target groups that can provide data on vaccination coverage, acceptance, and intention to be vaccinated.

With the data from school entry examinations and health claims, vaccination coverage can only be reported with a delay of 1–2 years. Furthermore, the health claims data are incomplete due to the increasing diversification of vaccination service providers. The need for a complete and timely monitoring of the vaccination coverage during the COVID-19 pandemic revealed the limitations of these systems in an emergency situation. Therefore, the temporary Digital Immunization Monitoring (DIM) system for collecting COVID-19 vaccination data was established. With DIM, it was possible to collect COVID-19 vaccination data from all vaccination service providers on a daily basis.

With the vision of timely and complete monitoring of vaccination coverage, the integration of vaccination data collection into the German Electronic Reporting and Information System for Infection Protection (DEMIS) is being developed as a uniform reporting infrastructure. In the future, DEMIS will be used to collect timely, pseudonymized, and individual-based vaccination data from all vaccination service providers. This will enable the evaluation of vaccination programs with little delay using complete and high-resolution data as well as the rapid adaptation of vaccination recommendations and communication strategies as needed.

## Hintergrund

Effektive Impfprogramme sind erforderlich, um die Bevölkerung vor impfpräventablen Erkrankungen und ihren Folgen optimal zu schützen. Um die Effektivität zu gewährleisten, sind für Evaluation und Anpassung aktuelle und qualitativ hochwertige Daten essenziell [1]. Dabei sind Impfquoten von herausragender Bedeutung [2]. Sie beschreiben den Anteil der Bevölkerung, der eine Impfung in Anspruch genommen hat, in Bezug auf die Bevölkerungsgruppe, der die Impfung empfohlen wird [3]. Impfquoten dienen damit sowohl der Evaluation der Leistung des Impfsystems als auch der Beurteilung der Akzeptanz von Impfangeboten in der Bevölkerung.

Zeitnah verfügbare und hochwertige Daten zu Impfquoten bilden außerdem eine wichtige Grundlage für strategische Entscheidungen in Impfprogrammen, damit Impfangebote präzise gesteuert und Ressourcen optimal eingesetzt werden können [4]. So können je nach Impfung die Impfquoten regional stark variieren und auch auf Unterschiede im Zugang zu Impfangeboten oder Informationen hinweisen [5]. Für die Berechnung von Impfquoten kommen verschiedene Datenquellen in Betracht. Hierzu zählen u. a. Gesundheitsinformationssysteme, Register und Abrechnungssysteme, aber auch Reihenuntersuchungen, Haushalts‑, Telefon- und Onlinesurveys. Alle Datenquellen weisen unterschiedliche Stärken und Schwächen auf und ergänzen sich in der Regel gegenseitig [6].

Das Robert Koch-Institut (RKI) hat den gesetzlichen Auftrag, Impfquoten in Deutschland zu erfassen und zu berichten. Seit mehreren Jahrzehnten existieren hierfür effektive Erhebungssysteme. Im Folgenden sollen diese Systeme mit ihren jeweiligen Besonderheiten und Anwendungsgebieten vorgestellt und ein Ausblick auf zukünftige Entwicklungen gegeben werden.

## Impfquotenerhebung in Deutschland – aktueller Stand

### Schuleingangsuntersuchungen

Lange Zeit boten die Datenerhebungen der flächendeckend durchgeführten Schuleingangsuntersuchungen (SEU) die einzige Datenquelle für ein kontinuierliches und gesetzlich festgelegtes Monitoring bundesweiter Kinderimpfquoten. Gemäß dem Infektionsschutzgesetz (IfSG) werden seit dem Jahr 2001 die SEU von den Gesundheitsämtern oder durch die von ihnen beauftragten Ärztinnen und Ärzte durchgeführt. Dabei werden Kinder vor der Aufnahme in die erste Klasse einer allgemeinbildenden Schule untersucht. Die Untersuchungsdaten werden auf Landesebene zentral gesammelt und ausgewertet. Manche Bundesländer erfassen zusätzlich soziodemografische Angaben, die mit der Nutzung von Impfungen in Zusammenhang stehen können. Die Daten zum Impfstatus werden dem RKI in aggregierter Form über einen standardisierten Erhebungsbogen übermittelt, der an die jeweils gültigen Empfehlungen der Ständigen Impfkommission (STIKO) angepasst ist. Auf dieser Datengrundlage berechnet das RKI Impfquoten und veröffentlicht die Ergebnisse auf bundesweiter Ebene und nach Bundesland aufgeschlüsselt [7].

Einer der wesentlichen Vorteile der SEU ist, dass jährlich der Impfstatus einer nahezu vollständigen Geburtskohorte ermittelt werden kann. Dabei wird die Anzahl entsprechend geimpfter Kinder anhand der vorgelegten Impfdokumentation (üblicherweise der Impfausweis) überprüft. Die Untersuchung bietet zudem Interventionsmöglichkeiten, wie direktes Impfen oder ein Informationsangebot über individuelle, noch erforderliche Nachholimpfungen. Der Anteil der Kinder mit vorgelegter Impfdokumentation beträgt in der Regel mehr als 90 % [7]. Die dokumentierten Impfungen werden auf die Gesamtzahl der Kinder, die ein Impfdokument vorzeigen, bezogen. Vermutlich unterliegen daher die Ergebnisse zu Impfquoten aus den SEU einer leichten Überschätzung [8, 9]. Andere Untersuchungen zeigen allerdings, dass nur geringe Unterschiede im Impfstatus zwischen den Gruppen mit und ohne Impfausweis bestehen [10].

Zu den Nachteilen der SEU gehört, dass das Alter der Kinder zum Zeitpunkt der SEU mit gewöhnlich 4 bis 6 Jahren deutlich über dem Altersbereich liegt, in dem die untersuchten Impfungen empfohlen werden. Damit ergibt sich bei der Evaluation der Impfakzeptanz ein Zeitverzug von mehreren Jahren. Aus dem gleichen Grund kann die Einführung neuer, für das Kleinkindalter vorgesehener Impfungen mit den Daten der SEU erst einige Jahre später nachvollzogen werden, wenn die Kinder das Schuleingangsalter erreicht haben. Nicht in den SEU untersucht werden können darüber hinaus Impfungen des Jugendlichen- bzw. Erwachsenenalters, also Impfungen, die jenseits des Einschulungsalters empfohlen sind. In den meisten Bundesländern und der zentralen Auswertung am RKI sind außerdem Informationen zum Impfalter sowie zu erforderlichen zeitlichen Abständen aufeinanderfolgender Impfstoffdosen einer Impfserie nicht Teil der Auswertungen. Es vergehen in der Regel 2 Jahre, bis die Daten erhoben, verarbeitet und dem RKI vollständig von allen Bundesländern übermittelt wurden und die Ergebnisse veröffentlicht werden können. Durch die Covid-19-Pandemie war die Durchführbarkeit der SEU außerdem stark eingeschränkt. Unvollständige und aufgrund selektiver Einladungen zu den SEU möglicherweise verzerrte Daten führten zum Ausfall der Veröffentlichung bundesweiter Ergebnisse der in den Jahren 2021 und 2022 durchgeführten SEU.

### Abrechnungsdaten der Kassenärztlichen Vereinigungen (KV-Impfsurveillance)

In Ergänzung zu den Analysen der SEU-Daten betreibt das RKI seit dem Jahr 2004 ein bundesweites Impfmonitoring, dass sich auf die Auswertung von Abrechnungsdaten aller 17 Kassenärztlichen Vereinigungen (KVen) stützt. Das zunächst vom RKI als Forschungsprojekt begonnene und später vom Bundesministerium für Gesundheit (BMG) weiterfinanzierte Vorhaben wurde im Jahr 2020 im IfSG gesetzlich verankert, mit dem Auftrag zur „Feststellung der Inanspruchnahme von Schutzimpfungen und von Impfeffekten“.

Grundlage dieser „KV-Impfsurveillance“ (KVIS) sind Abrechnungsdaten aus der vertragsärztlichen Versorgung, die die ambulante Versorgung der gesetzlich Krankenversicherten (ca. 87 % der Bevölkerung) widerspiegeln. Im Rahmen der quartalsweisen Abrechnung übermitteln die Vertragsarztpraxen ihre Daten an die jeweils zuständige KV. Der für die KVIS relevante Datenauszug wird von den KVen über eine bereitgestellte Anwendung weiterverarbeitet und dem RKI pseudonymisiert übermittelt. Zu den bereitgestellten Datensätzen zählen ausgewählte Daten zu abgerechneten Impfleistungen, Vorsorgeuntersuchungen und Abrechnungsdiagnosen. Die Variablen umfassen insbesondere das Patientenpseudonym, Geburtsmonat und -jahr, das Geschlecht, die Postleitzahl des Wohnortes und davon ableitbar den Kreis, das Leistungsdatum, den ICD-10-Erkrankungscode gemäß der Internationalen Klassifikation der Krankheiten, das Quartal der Diagnose sowie die Abrechnungsziffern der Impfungen, die die Zuordnung zur jeweils verimpften Antigenkombination erlauben.

Innerhalb einer KV-Region werden die Patientinnen und Patienten mit einem über die Zeit einheitlichen Verfahren pseudonymisiert. Jede KV nutzte bisher ein KV-spezifisches Pseudonymisierungsgeheimnis. Damit waren alle individuellen Patientendaten innerhalb einer KV verknüpfbar, KV-übergreifend allerdings nicht. Die Auswertungen wurden daher in den meisten Fällen innerhalb von KV-spezifischen Untersuchungskohorten durchgeführt, bei denen über verschiedene Einschlusskriterien sichergestellt wurde, dass eine vollständige Verknüpfung aller vorhandenen Daten zu einer Patientin oder einem Patienten unterbrechungsfrei über längere Zeiträume von Monaten oder Jahren möglich ist. Innerhalb dieser Kohorten werden die individuellen Impfhistorien rekonstruiert und daraus die Impfquoten der empfohlenen Kinder‑, Jugend- und Erwachsenenimpfungen berechnet [7, 11].

Die KVIS bietet viele Vorteile für die Analyse des Impfgeschehens, die weit über die Möglichkeiten der SEU hinausgehen. So ist die Auswertung sowohl in Altersquerschnitten (z. B. Geburtsjahrgängen) und als zeitliche Längsschnittanalysen (z. B. als Vergleich von Kalenderjahren) möglich. Neben der bundesweiten Aggregation sind auch kleinräumigere Auflösungen bis auf Kreisebene sowie Aufschlüsselungen nach Geschlecht und Alter möglich. Gleichzeitig erlauben die Daten eine Analyse der Impfabstände sowie der altersgerechten Inanspruchnahme von Impfungen. Damit lässt die KVIS neben einer Gesamtevaluierung von Impfempfehlungen auch detaillierte Betrachtungen der Umsetzung geänderter Impfschemata zu. Als Beispiele seien hier der Übergang auf das reduzierte Impfschema der Pneumokokkenimpfung und der Sechsfachimpfung sowie das verkürzte Impfschema gegen humane Papillomviren in jüngeren Zielgruppen genannt. Die in der KVIS verarbeiteten Daten zu Diagnosen und Vorsorgeuntersuchungen enthalten Informationen zur Einordnung der Personen mit Impfindikation und der Berechnung der Impfinanspruchnahme in diesen Gruppen (z. B. Pneumokokkenimpfquote bei Personen mit impfrelevanten Grunderkrankungen; Influenzaimpfquote bei Schwangeren). Da auch Diagnosedaten zu den Zielkrankheiten der Impfungen vorliegen, sind eine Berechnung von Erkrankungshäufigkeiten der zu verhindernden Erkrankungen und Untersuchungen zur möglichen Änderung ihrer Epidemiologie nach breiter Nutzung der Impfung in der Bevölkerung durchführbar [12, 13]. Durch die vergleichende Analyse von Erkrankungshäufigkeiten in geimpften und ungeimpften Gruppen können zudem Aussagen über die Impfeffektivität und die Schutzdauer getroffen werden [14].

Auch in der KVIS liegen die Daten erst mit einem Zeitverzug vor, jedoch nicht so verzögert wie bei den SEU. Der Zeitverzug beträgt in der Regel 2–3 Quartale. Dies ist ein Nachteil der Methode bei der Bereitstellung aktueller Ergebnisse, die insbesondere für die Bewertung saisonaler Impfaktivitäten (z. B. Impfung gegen Influenza oder Covid-19) notwendig sind. Darüber hinaus werden nicht von allen Akteurinnen und Akteuren im Impfregelsystem die Leistungen in den Abrechnungsdaten der KVen erfasst. Es fehlen beispielsweise Daten aus der Betriebsmedizin, den Apotheken und dem Öffentlichen Gesundheitsdienst (ÖGD) sowie Leistungen, die im Bereich der Hausarztzentrierten Versorgung (HzV) erbracht werden. Das Fehlen dieser Daten kann die in der KVIS bereitgestellten Ergebnisse verzerren. In den SEU erfolgen die Untersuchungen unabhängig vom Versichertenstatus, hingegen können in der KVIS keine Aussagen zu Privatversicherten getroffen werden. Außerdem erlauben die SEU die Datenerhebung von nach Deutschland hinzugezogenen Kindern, während sich in der KVIS ihre Impfhistorien nur unvollständig abbilden lassen. Trotz dieser Unterschiede zwischen beiden Datenquellen konnten die Ergebnisse der SEU und KVIS in vergleichenden Analysen zur Impfinanspruchnahme erfolgreich validiert werden [15]. Im Gegensatz zur SEU bietet die KVIS allerdings keine Interventionsmöglichkeiten. Eine Re-Identifikation der Personen für eine individuelle Impferinnerung ist in der KVIS weder möglich noch räumt der Gesetzgeber diese Möglichkeit ein.

Sowohl die Ergebnisse der SEU als auch der KVIS zur Impfinanspruchnahme sind Teil der internationalen Gesundheitsberichterstattung des RKI und werden jährlich an die Weltgesundheitsorganisation (WHO) und dem Kinderhilfswerk der Vereinten Nationen (UNICEF) übermittelt [16].

### Digitales Impfquotenmonitoring

Die Einführung wirksamer Covid-19-Impfstoffe im Dezember 2020 galt als ein entscheidender Faktor für die Eindämmung der Covid-19-Pandemie. Valide und zeitnah verfügbare Daten zur Inanspruchnahme der Covid-19-Impfung waren essenziell, um den Stand der Impfkampagne zu jedem Zeitpunkt einzuschätzen und die Wirksamkeit und Sicherheit der neuen Impfstoffe im breiten Bevölkerungseinsatz fortlaufend zu bewerten. Die KVIS als Kernstück des etablierten Routine-Impfmonitorings genügte den Anforderungen an ein pandemisches Impfmonitoring mit einer aktuellen und vollständigen Impfdatenerhebung nicht, da die Daten in der KVIS erst mit einem großen zeitlichen Abstand zum Impfzeitpunkt vorliegen. Zudem begann die Covid-19-Impfkampagne außerhalb des Routine-Impfsystems in von den Bundesländern eingerichteten Impfzentren und mittels mobiler Impfteams.

Um eine aktuelle und vollständige Erhebung der Covid-19-Impfdaten sicherzustellen, war es notwendig, eine neue Struktur der Impfdatenerhebung aufzubauen. Mit dem „Digitalen Impfquotenmonitoring“ (DIM) wurde in kürzester Zeit ein neues Meldesystem entwickelt, über das alle an der Impfkampagne teilnehmenden Stellen tagesaktuelle Impfdaten an das RKI übermitteln konnten [17, 18]. Die Meldewege und Datenstrukturen für die jeweiligen Leistungserbringer waren per Rechtsverordnung verbindlich vorgegeben (§ 4 Coronavirus-Impfverordnung bzw. § 3 COVID-19-Vorsorgeverordnung). Das DIM bestand aus 3 temporär eingerichteten Übermittlungssystemen:

- Impfstellen, die nicht zum Routine-Impfsystem gehörten (Impfzentren, mobile Impfteams, Krankenhäuser, Betriebsmedizin, Apotheken, ÖGD und Zahnarztpraxen), nutzten das vom RKI in Zusammenarbeit mit der Bundesdruckerei-Gruppe GmbH (bdr) entwickelte Erhebungssystem zur Übermittlung pseudonymisierter Einzelfalldaten (DIM-Portal). Das DIM-Portal war zum Start der Impfkampagne einsatzbereit. Im Zuge mehrmaliger Aktualisierungen der Impfverordnung wurden sukzessive weitere Einrichtungen als Leistungserbringer an das DIM-Portal angebunden.
- Die Kassenärztliche Bundesvereinigung (KBV) stellte ein Meldeportal für alle Vertragsärztinnen und -ärzte zur Übermittlung tagesbasierter aggregierter Datenmeldungen bereit.
- Der Verband der Privatärztlichen Abrechnungsstellen (PVS) stellte ein Meldeportal für alle Privatärztinnen- und -ärzte zur Übermittlung tagesbasierter aggregierter Datenmeldungen bereit.

Das RKI wertete die Impfdaten aus und publizierte die Ergebnisse. Auswertungen zum Impffortschritt in der Gesamtbevölkerung, in den verfügbaren Altersgruppen und nach Region sowie zu eingesetzten Impfstoffen wurden u. a. auf der RKI-Website publiziert, auf dem Impfdashboard (https://www.impfdashboard.de) des BMG visualisiert und in digitaler Form auf dem Datenrepositorium GitHub [17] bereitgestellt. Die Impfquoten waren auch die Basis für eine kontinuierliche Bewertung der Impfeffektivität, die regelmäßig in den Wochen- bzw. Monatsberichten des RKI veröffentlicht wurde [19], sowie Grundlage für epidemiologische Untersuchungen, z. B. zu Impfeffekten [20]. Weiterhin wurden Impfdaten für RKI-interne Modellierungen und von der STIKO zur Evaluation ihrer Empfehlungen genutzt. Zusätzlich wurden diese Daten dem Europäischen Surveillancesystem TESSy und dem Paul-Ehrlich-Institut für die Überwachung der Impfstoffsicherheit zur Verfügung gestellt sowie für externe Anfragen u. a. aus Politik, Medien und dem ÖGD aufbereitet.

Limitationen in der Datenauswertung bestanden insbesondere durch die geringe Granularität der Daten aus den Meldeportalen der niedergelassenen Ärzteschaft, die bundesweit mehr als die Hälfte aller gemeldeten Impfungen ausmachten. Über das Impfgeschehen konnte daher nur in groben Aggregationsstufen zuverlässig berichtet werden. Der Impffortschritt in differenzierteren Altersgruppen, nach Geschlecht oder für spezifische Personengruppen (z. B. Personen mit Grunderkrankung, Schwangere) war mit den verfügbaren Daten nicht darstellbar und stellte eine wesentliche Limitation dar, insbesondere im Kontext der Pharmakovigilanz. Auch konnten keine Impfhistorien auf individueller Ebene abgebildet werden. Eine konsistente regionale Zuordnung zum Wohnort der Geimpften war ebenfalls nicht möglich.

Für die Steuerung und Evaluation der Covid-19-Impfkampagne waren aktuelle und belastbare Daten zum Immun- und Impfstatus der Bevölkerung unerlässlich. Die Impfquoten aus DIM bildeten eine wichtige Grundlage für die Evaluation der Umsetzung der Impfempfehlungen und für politische Entscheidungen zum Impfgeschehen während der Pandemie. Insgesamt wurden 197 Mio. durchgeführte Covid-19-Impfungen (davon 89,7 Mio. über das DIM-Portal) im Rahmen der Laufzeit des DIM berichtet. Mit dem Übergang in ein endemisches Geschehen und der Überführung der Covid-19-Impfung in die Regelversorgung war es nicht weiter notwendig, die neu eingerichteten Erhebungsstrukturen des DIM aufrechtzuerhalten. Das DIM-Portal wurde zum 31.03.2024 und das Meldeportal für die niedergelassene Vertragsärzteschaft zum 30.06.2024 geschlossen und die gesonderte Berichterstattung zur Covid-19-Impfung eingestellt. Zukünftig wird die Covid-19-Impfung wie alle von der STIKO empfohlenen Routineimpfungen im Rahmen der KVIS erhoben und ausgewertet.

Um die Covid-19-Impfdaten aus dem DIM-Portal für weiterführende Datenauswertungen nachnutzen zu können, musste die Verknüpfbarkeit dieser Daten mit den KV-Daten sichergestellt werden. Daher wurden die KV-Daten retrospektiv ab dem ersten Abrechnungsquartal 2020 bis einschließlich dem ersten Abrechnungsquartal 2024 über die bdr nach DIM-Verfahren pseudonymisiert. Mit den zusammengeführten KV- und DIM-Daten werden Auswertungen u. a. zu differenzierten Impfquoten nach Alter, Geschlecht, Risikogruppe und Region sowie zur Impfeffektivität oder zu Post-Covid möglich.

### Bevölkerungssurveys

Um wirksame Strategien entwickeln zu können, mit denen mehr Menschen erreicht und vor impfpräventablen Krankheiten geschützt werden können, ist es auch essenziell zu verstehen, wie viele, welche und warum Menschen sich *nicht* impfen lassen. Daher nutzt das RKI neben den beiden etablierten Säulen des Impfquotenmonitorings (KVIS und SEU) und dem zeitweise eingesetzten DIM auch Surveys für die Erfassung von Daten zum Impfstatus, zur Impfakzeptanz und -intention. Diese Surveys bilden eine unabdingbare Ergänzung, um soziodemografische und -ökonomische Muster zu erkennen und die behavioralen Hintergründe von Impfentscheidungen zu verstehen. Sie erfassen deutlich mehr Informationen, als die Abrechnungsdaten der Ärzteschaft und die Meldedaten aus DIM liefern können, insbesondere bezogen auf Zielgruppen, die nicht in den o. g. Systemen identifiziert werden können (z. B. Impfquoten bei medizinischem Personal oder Menschen mit niedrigem sozioökonomischen Status). So können förderliche Faktoren sowie Barrieren der Impfinanspruchnahme identifiziert werden. Die Surveydaten ermöglichen damit ein umfassenderes Verständnis des Impfgeschehens in Deutschland und können Ansatzpunkte für gezielte Maßnahmen zur Schließung von Impflücken aufzeigen.

Surveys zu Impfakzeptanz und -intention wurden in der Vergangenheit und auch aktuell zumeist sporadisch und anlassbezogen durchgeführt, z. B. Befragungen von Gesundheitspersonal und Eltern zur HPV-Impfung im Rahmen der Interventionsstudie zur Steigerung der HPV-Impfquoten in Deutschland (InveSt HPV; [21]). Im Rahmen des bundesweiten Gesundheitsmonitorings des RKI wurden in Abständen von mehreren Jahren wiederholt Querschnittsdaten zur Gesundheit von Kindern und Jugendlichen (KiGGS) sowie von Erwachsenen (GEDA) erhoben. Damit konnten u. a. Aussagen zu Trends sowie zum Zusammenhang zwischen Impfstatus und soziodemografischen Parametern getroffen werden [22]. Die Onlinebefragung von Klinikpersonal zur Influenzaimpfung (OKaPII) untersucht seit 2017 die Impfinanspruchnahme und -akzeptanz in der speziellen Zielgruppe in Form von jährlich wiederholten Querschnittsbefragungen [23, 24]. Während der Covid-19-Pandemie wurde am RKI das COVID-19 Impfquoten-Monitoring in Deutschland (COVIMO) zur Evaluation der Covid-19-Impfakzeptanz und -intention etabliert und es konnten relevante Empfehlungen zur Steigerung der Covid-19-Impfquoten in der Gesamtbevölkerung sowie speziellen Zielgruppen ausgesprochen werden [25, 26]. Aktuell befindet sich am RKI das Panel „Gesundheit in Deutschland“ im Aufbau, das fortlaufend qualitativ hochwertige und für Deutschland repräsentative Daten zu Gesundheitsfragen liefern soll. Darin sollen u. a. die bisherigen Monitoringstudien KiGGS und GEDA aufgehen. Das Studiendesign sowie die geplanten Erhebungswellen sind an anderer Stelle beschrieben [27]. Mit einer neuen Befragungsinfrastruktur können zukünftig Daten zum Impfverhalten im Zusammenspiel mit sozialen Merkmalen untersucht und Trends analysiert werden.

## Aktuelle Entwicklungen und Perspektive

### Neuauflage VacMap und neues Berichtsformat

Bis zum Jahr 2022 publizierte das RKI einmal jährlich erscheinende Impfquotenberichte im Epidemiologischen Bulletin, differenziert nach Altersgruppe und Region sowie im Zeitverlauf [7, 11]. Dieses ausführliche Berichtsformat wurde im Jahr 2024 durch einen auf die aktuellen und wesentlichen Entwicklungen fokussierten Kurzbericht abgelöst. Gleichzeitig erfolgt die Publikation von Impfquoten aus der KVIS auf dem Impfdashboard VacMap, das im Dezember 2023 neu gestaltet wurde und kontinuierlich weiterentwickelt und regelmäßig aktualisiert wird [28]. Zudem können über GitHub die Gesamtdatensätze heruntergeladen und nachgenutzt werden [29].

**Abb. 1:**
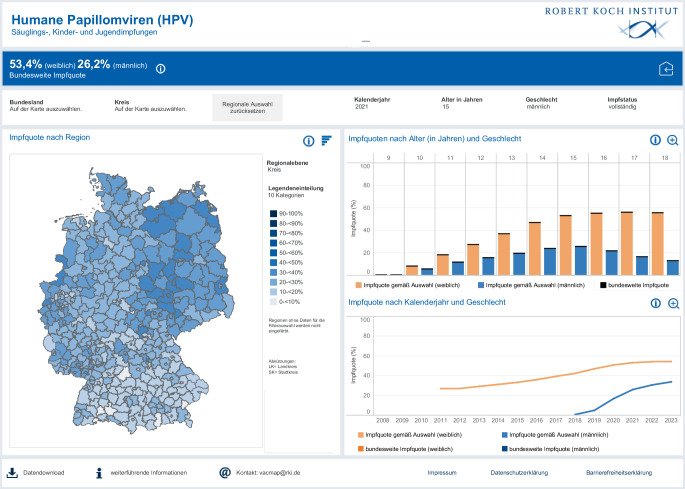
VacMap – Dashboard zum Impfgeschehen. Das Dashboard zeigt die Inanspruchnahme von Impfungen nach Region, Zeit, Alter und Geschlecht. Hier am Beispiel der Impfung gegen humane Papillomviren. (Eigener Screenshot von [28])

Mit VacMap werden die Ergebnisse der KVIS für Akteurinnen und Akteure der Impfprävention und die breite Öffentlichkeit anschaulich aufbereitet und niedrigschwellig öffentlich zugänglich gemacht. VacMap visualisiert die jeweils aktuellen, nach Region und Altersgruppe aufgeschlüsselten Impfquoten (Abb. 1) und erlaubt so, den Grad der Umsetzung von Impfempfehlungen abzuschätzen und lokale oder altersabhängige Lücken bei der Inanspruchnahme einzelner Impfungen zu identifizieren sowie Maßnahmen zur Steigerung der Impfinanspruchnahme zielgerichtet zu planen.

### Vision einer aktuellen und vollständigen Impfdatenerhebung

Die KV-Abrechnungsdaten werden auch zukünftig wesentliche Grundlage des Impfquotenmonitorings in Deutschland sein. Jedoch spielen neben der vertragsärztlichen Versorgung mit Abrechnung über die KVen zunehmend weitere Leistungserbringer bei der Erbringung von Impfleistungen eine Rolle. Dazu zählen insbesondere Apotheken, die Betriebsmedizin, der ÖGD sowie die neben den KVen existierende Struktur der HzV. So werden beispielsweise Influenza- oder Masernimpfungen häufig auch im betrieblichen Kontext verabreicht, können aber bislang nicht in den Auswertungen des RKI berücksichtigt werden. Da rund 10 % der gesetzlich Krankenversicherten an Verträgen der HzV teilnehmen und der Versorgungsgrad durch HzV-Verträge regional variiert, wird ein zunehmender Einfluss auf die Validität der Datenanalysen in der KVIS wahrscheinlich. Aus dem Bereich der privaten Krankenversicherung liegen mit Ausnahme zur Covid-19-Impfung bislang keine Daten vor. Mit der Erweiterung des § 13 Absatz 5 IfSG zur Impfsurveillance im September 2022 wurde die Rechtsgrundlage für die Meldepflicht aller durchgeführten Impfungen für alle impfenden Stellen geschaffen.

Die Notwendigkeit eines vollständigen und zeitnahen Impfquotenmonitorings während der Covid-19-Pandemie zeigte die Grenzen des bisherigen Impfmonitoringsystems der KVIS auf. Unter hohem Zeitdruck und Arbeitsaufwand konnte mit dem DIM-Portal eine digitale Lösung geschaffen werden, die es Impfstellen außerhalb des Routine-Impfsystems ermöglichte, ihrer Meldepflicht nachzukommen, und in Kombination mit den Meldeportalen der niedergelassenen Ärzteschaft die Erhebung aktueller und belastbarer Impfdaten zur Evaluation und Steuerung der Covid-19-Impfkampagne erlaubte. Die Erweiterung des DIM-Portals im Herbst 2023 um die Meldung von Grippeschutzimpfungen ermöglichte zusätzlich die Übermittlung von Influenzaimpfungen aus den DIM-Meldestellen in der Saison 2023/2024. Seit der Abschaltung des DIM-Portals besteht aktuell für die ehemaligen DIM-Melder jedoch keine technische Möglichkeit mehr, Impfdaten an das RKI zu übermitteln.

**Abb. 2:**
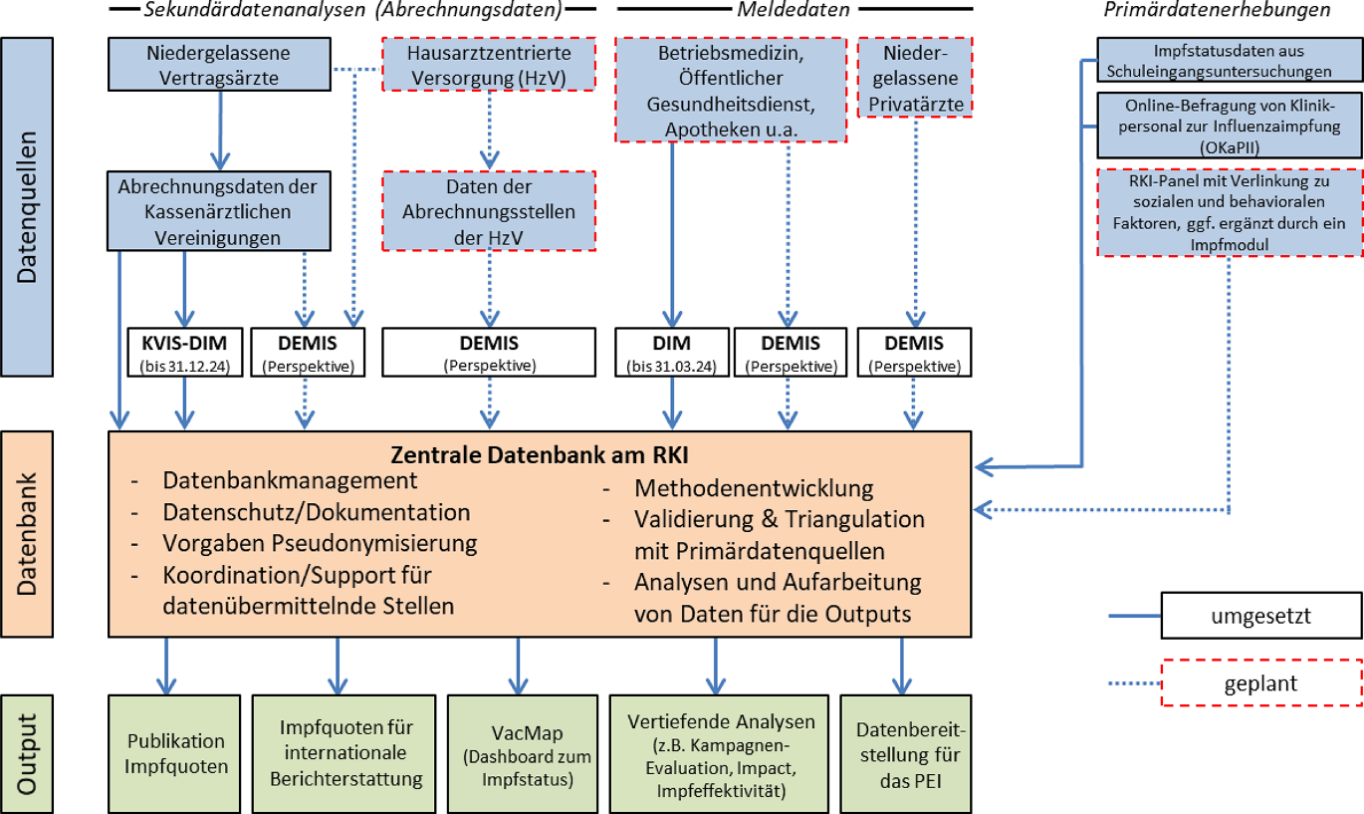
Datenfluss im Impfquotenmonitoring in Deutschland – gegenwärtig und perspektivisch. *DEMIS* Deutsches Elektronisches Melde- und Informationssystem für den Infektionsschutz; *DIM* Digitales Impfquotenmonitoring; *HzV* Hausarztzentrierte Versorgung; *KV* Kassenärztliche Vereinigung; *KVIS* KV-Impfsurveillance; *PEI* Paul-Ehrlich-Institut; *RKI* Robert Koch-Institut. Die Abbildung gibt einen Überblick über die aktuellen und geplanten Datenflüsse im Impfquotenmonitoring. Die Impfdatenerhebung wird perspektivisch in eine einheitliche Meldeinfrastruktur integriert. Für die Übermittlung zeitnaher Covid-19-Impfdaten aus Impfstellen außerhalb des niedergelassenen Bereiches wurde das DIM bis zum 31.03.2024 betrieben. Die Daten wurden über die Bundesdruckerei pseudonymisiert. Die KV-Abrechnungsdaten wurden bisher in pseudonymisierter Form direkt dem RKI übermittelt. Zusätzlich durchliefen diese Daten bis zum 31.12.2024 retrospektiv ab Abrechnungsjahr 2020 das DIM-Pseudonymisierungsverfahren, um die DIM- mit den KV-Daten verknüpfen zu können. Die Anbindung der KVen und der HzV an DEMIS ist eine notwendige Übergangsphase, bis die niedergelassenen Ärztinnen und Ärzte langfristig durch Schnittstellen zu den entsprechenden Praxisinformationssystemen ihre Impfdaten aus der Praxis automatisiert via DEMIS übermitteln können. Im RKI wird die Verknüpfbarkeit der Daten aus allen Quellen sichergestellt. Die Daten werden einer integrierten Auswertung zugeführt. (Eigene Abbildung)

Vor dem Hintergrund dieser Erfahrungen soll es zukünftig über die Infrastruktur des Deutschen Elektronischen Melde- und Informationssystems für den Infektionsschutz (DEMIS) eine langfristige Lösung zur zeitnahen Übermittlung von Impfdaten von allen Impfleistungserbringern geben (Abb. 2). Mit DEMIS wurde bundesweit eine Infrastruktur zur elektronischen Meldung von meldepflichtigen Erregernachweisen und Krankheiten gemäß IfSG aufgebaut, in die perspektivisch neben weiteren Surveillancesystemen auch das DIM-Portal integriert werden soll (https://wiki.gematik.de/display/DSKB).

Der Ausbau von DEMIS als einheitliche Meldeplattform für Impfdaten soll schrittweise erfolgen. Das dabei eingesetzte einheitliche Pseudonymisierungsverfahren ermöglicht zum einen die Verknüpfbarkeit der Daten aller meldenden Stellen auf individueller Ebene, zum anderen verhindert die Patientenverschlüsselung eine Re-Identifizierung der Personen. Zunächst werden die ehemaligen DIM-Melder technisch dazu befähigt, Impfdaten über DEMIS zu übermitteln. Hierfür werden in der DEMIS-Umgebung ein Meldeportal sowie eine Schnittstelle zur Übermittlung von Impfdaten bereitgestellt. Parallel werden die KVen angebunden, um sicherzustellen, dass die über DEMIS übermittelten Impfdaten der ehemaligen DIM-Melder mit den KV-Daten für Auswertungen verknüpfbar sind. Die Integration der HzV in die Impfsurveillance kann aufgrund derselben Datenstruktur analog zu dem Vorgehen bei den KVen erfolgen. Die Anbindung der KVen/HzV an DEMIS ist eine notwendige Übergangsphase, bis die niedergelassenen Ärztinnen und Ärzte langfristig durch Schnittstellen zu den entsprechenden Praxisinformationssystemen ihre Impfdaten aus der Praxis automatisiert und ohne relevante Mehrarbeit für das medizinische Personal via DEMIS übermitteln können. Erst mit diesem letzten Schritt wird die Vision einer aktuellen und vollständigen Erhebung von Impfdaten in Deutschland Realität.

Das Panel „Gesundheit in Deutschland“ bietet außerdem die Chance, perspektivisch ein Impfakzeptanzmonitoring am RKI zu etablieren. Damit können auch behaviorale Merkmale in Bezug auf das Impfverhalten sowie die Impfbereitschaft repräsentativ untersucht werden, um zeitnah und fortlaufend ein tiefergehendes Verständnis zu gewinnen und bei Bedarf Maßnahmen zur Impfquotensteigerung zu ergreifen.

Um Impflücken wirksam zu schließen und Impfquoten zu erhöhen, sollte in Zukunft auch die Möglichkeit der elektronischen Patientenakte (ePA) genutzt werden. So sollte Versicherten durch die Integration von Impferinnerungen im elektronischen Impfpass die Möglichkeit gegeben werden, sich niederschwellig an ausstehende Impfungen erinnern zu lassen. Aufgrund der *Opt-out* Regelung erscheint die Nutzung der ePA für die Erhebung repräsentativer Impfquoten dagegen ungeeignet.

## Fazit

Aktuelle und belastbare Impfdaten sind entscheidend für den Erfolg nationaler Impfprogramme. In Deutschland wurden in den letzten Jahrzehnten mit den SEU und der KVIS zwei wertvolle Monitoringsysteme aufgebaut, die während der Covid-19-Pandemie um tagesaktuelle Covid-19-Impfdaten aus dem DIM-System ergänzt wurden. In Zukunft wird mit der geplanten Integration der Impfdatenerhebung in eine einheitliche Meldeinfrastruktur ein vollständiges und zeitnahes Impfmonitoring auf Bevölkerungsebene in Deutschland angestrebt. Damit wird es möglich sein, die Umsetzung von Impfempfehlungen und die Einführung neuer Impfstoffe anhand vollständiger und hoch aufgelöster Daten mit geringem Zeitverzug zu bewerten und letztendlich die Bevölkerung vor impfpräventablen Erkrankungen wirksamer zu schützen. Die Nutzung solcher Daten für die Bewertung von Impfeffektivität und Impfstoffsicherheit trägt zum Vertrauen in Impfungen und in das Impfsystem in Deutschland bei.
